# A case study of HIV/AIDS services from community-based organizations during COVID-19 lockdown in China

**DOI:** 10.1186/s12913-023-09271-4

**Published:** 2023-03-27

**Authors:** Jennifer Z.H. Bouey, Jing Han, Yuxuan Liu, Myriam Vuckovic, Keren Zhu, Kai Zhou, Ye Su

**Affiliations:** 1 Department of Global Health, School of Health, Georgetown University, 3700 Reservoir Road NW, Washington, DC 20057 USA; 2 RAND Corporation, Santa Monica, CA USA; 3 Ditan Infectious Disease Hospital, Beijing, China; 4 UNAIDS China, Beijing, China; 5 Home of Red Ribbon, Beijing, China

**Keywords:** COVID-19, HIV/AIDS care, Community-based Organizations, Telemedicine, Community capacity building

## Abstract

**Introduction:**

People living with HIV (PLHIV) relied on community-based organizations (CBOs) in accessing HIV care and support during the COVID-19 pandemic in China. However, little is known about the impact of, and challenges faced by Chinese CBOs supporting PLHIV during lockdowns.

**Methods:**

A survey and interview study was conducted among 29 CBOs serving PLHIV in China between November 10 and November 23, 2020. Participants were asked to complete a 20-minute online survey on their routine operations, organizational capacity building, service provided, and challenges during the pandemic. A focus group interview was conducted with CBOs after the survey to gather CBOs’ policy recommendations. Survey data analysis was conducted using STATA 17.0 while qualitative data was examined using thematic analysis.

**Results:**

HIV-focused CBOs in China serve diverse clients including PLHIV, HIV high-risk groups, and the public. The scope of services provided is broad, ranging from HIV testing to peer support. All CBOs surveyed maintained their services during the pandemic, many by switching to online or hybrid mode. Many CBOs reported adding new clients and services, such as mailing medications. The top challenges faced by CBOs included service reduction due to staff shortage, lack of PPE for staff, and lack of operational funding during COVID-19 lockdowns in 2020. CBOs considered the ability to better network with other CBOs and other sectors (e.g., clinics, governments), a standard emergency response guideline, and ready strategies to help PLHIV build resilience to be critical for future emergency preparation.

**Conclusion:**

Chinese CBOs serving vulnerable populations affected by HIV/AIDS are instrumental in building resilience in their communities during the COVID-19 pandemic, and they can play significant roles in providing uninterrupted services during emergencies by mobilizing resources, creating new services and operation methods, and utilizing existing networks. Chinese CBOs’ experiences, challenges, and their policy recommendations can inform policy makers on how to support future CBO capacity building to bridge service gaps during crises and reduce health inequalities in China and globally.

## Introduction

The COVID-19 pandemic is a defining catastrophic public health event of our lifetimes.In China, the COVID-19 pandemic started with a national lockdown in 2020. After two years of conservative intervention focusing on mass testing and quarantine, COVID-19 came back in 2022 and caused millions of infections and an overwhelming death toll, which put a significant strain on China’s healthcare system and paralyzed the economic engine. How do Chinese community organizations support vulnerable populations such as HIV/AIDS patients during such disasters? What policy changes do the Chinese CBOs hope to see? Our case study strives to answer these questions.

China was the first country to encounter the novel coronavirus disease and to implement a strict, large-scale lockdown between January 23, 2020, and April 2020 to contain the virus [1, 2]. Most of the 31 provinces in China declared the highest Emergency Level on January 23rd of 2020, enabling local governments to employ social policing mechanisms to enforce quarantine and to close public events with crowd gatherings across the country. Most highways and public transportation were shut down (January 23- February 7) [2]. All businesses and recreational facilities were closed, except for medical emergency rooms, grocery stores, and keyinfrastructure-related economic activities. In rural areas, many villages stalled traffic and set up entrance checks, whereas urban residential communities required residents to prove their residence to use the weekly grocery shopping quota [3]. After the outbreak peaked in mid-February, many prefecture-level cities switched from the stringentshutdown to semi-lockdown for another month [4]. Wuhan, the capital of Hubei province and the origin of the outbreak, experienced the longest lockdown from January 21 to April 7, 2020 [5]. COVID-19 and the stringent intervention had a profound impact on the lives of the Chinese people during this initial period of the COVID-19 pandemic [6, 7].

China is also home to 1.05 million people living with HIV (PLHIV) who needed long-term antiviral medical treatment and care in 2020 [8]. The central government provides PLHIVs access to free antiretroviral therapy (ART) and free voluntary counseling and testing since 2003 [9]. In 2016, the new “Treatment for All’’ policy removed the requirement of low CD4 levels as a treatment qualifier. By 2020, about 978,138 PLHIV had gained access to ART, covering 92.9% of all PLHIV in China [8]. Despite this progress and updated policies, various structural, psychological, and behavioral barriers to ART adherence persist [10]. Barriers including patients’ concerns for side effects and “pill burden,” lack of effective communication between patients and health care providers, low patient self-efficacy of ART, competing priorities for patients, and depression and stigma associated with HIV [11, 12]. Like many Community-Based Organizations (CBO) serving vulnerable populations globally, Chinese CBOs play critical roles in helping PLHIV to improve their access to HIV screening, treatment and care, and reduce stigma, especially among men who have sex with men (MSM) [13–16]. In China, there are two main types of CBOs providing services to PLHIV: those independently registered with the state/provincial or local government of Civil Affairs as a non-profit organization, and those affiliated with health clinics and local public health offices without an independent registry [17]. Both types of CBOs rely on government public health agencies for funding.

COVID-19 has led to unprecedented stress on health and public health systems and has intensified disruptions in HIV prevention, testing, and HIV care continuum services worldwide [18]. China is not an exception. A Chinese provincial study based on the HIV registration system found a 49% drop in HIV testing rates and a 37% drop in new HIV diagnoses during the first months of COVID-19. In addition, only half of the 475 newly diagnosed HIV patients underwent CD4 count testing and 28.6% did not receive routine linkage to care in the same time period [19].

Around the world,PLHIV and high-risk populations rely on CBOs for their rich local knowledge, operational flexibility, and direct contact to people in need, to provide humanitarian aid during a crisis [20]. Facing challenges due to quarantine requirements and transportation service requirements, CBOs in many countries responded by moving their services online and utilizing technology-driven solutions to promote access to HIV counseling, testing, and treatment [21]. Global [22, 23] and China-specific [24] studies have shown that CBOs promoting community connectedness among MSM resulted in higher HIV testing rates during COIVD-19. However, CBOs themselves are not immune to the negative impact of COVID-19. Preliminary studies in the U.S. have found that the COVID-19 pandemic presents multifaceted challenges to CBOs providing HIV services, including but not limited to structural inequality, resources shortages, and disruption to patient-centered services provision [25, 26].

Clearly, more in-depth studies among CBOs are necessary to understand COVID-19’s impact among CBOs serving PLHIV in China. Although several studies in China highlighted the challenges to PLHIV [19, 27–29] and healthcare workers [30, 31], few looked at the implications for Chinese CBOs during the lockdowns. Only one stakeholder study published in English included 17 CBO workers in the interviews and found that CBOs could assist HIV care among PLHIV in multiple ways during COVID-19 [13].

In this study, a team of CBO leaders, clinicians, and public health researchers try to answer the following questions using data collected from a mixed-method study (survey and focus group): What challenges did PLHIV face during the first pandemic lockdown in China? What were the challenges to the CBOs and how did they cope? What innovation came out of the crisis? What gaps in CBOs’ capacity were revealed and how to build better preparedness for future emergencies? The study provides critical information on how best to prepare and utilize community organization services on HIV care during a public health emergency or a disaster.

## Methods

### Study design and participants

The study research team included HIV specialists of a large infectious disease hospital in Beijing, their affiliated and long-term CBO partner “Home of Red Ribbon (HRR),” staff of the Joint United Nations Programme on HIV/AIDS (UNAIDS) Beijing office, and global health researchers from Georgetown University and the RAND Corporation, an American think tank that develops solutions to public policy challenges. HRR was founded in 1999 to serve local PLHIV. In 2019, HRR founded the “Beijing Red Ribbon Alliance,” a national platform with 60 + CBO members serving PLHIV. In this study, CBO participants were recruited by HRR from the Alliance. They come from all seven geographic regions of China and were considered reliable information sources based on previous collaborations with HRR. Organizations participating in the study had to meet the following criteria: (a) established Chinese CBOs providing HIV prevention and treatment services; (b) delivered services from January 20, 2020, to April 29, 2020; and (c) would like to provide informed consent for the study. Among survey invitations to 32 qualified CBOs, 29 CBOs responded. Three CBOs have multiple branch offices that completed the survey, yielding a 90% response rate.

### Data collection

Both qualitative and quantitative data on service provision and needs during the COVID-19 pandemic were collected from the participating CBOs. A 20-minute online survey with both closed- and open-ended questions was first disseminated and collected between November 10 and November 23, 2020, and a virtual focus group interview was conducted on November 13, 2020. In addition to the survey data, detailed notes from the focus group interview were used for theme analysis. All data collection followed the principles of confidentiality and voluntary participation. There were no consequences if a participant withdrew from the study. The study protocol was approved by the Ditan Hospital Internal Research Board (IRB number: KY2020-019).

### Survey

The online survey questionnaire was designed by the research team staff from Beijing Ditan Hospital with HRR. A Chinese online survey platform “Wen-Juan-Xing’’ was used to host the survey, which was then disseminated to the invited participants through the social media app WeChat. The CBO organizations that completed the online survey received RMB 800 (approximately USD 125) for their participation. The questionnaire contained nineteen close-ended multiple-choice questions and eleven open-ended short-answer questions to cover the following three domains:

#### CBO routine operational characteristics

questions obtained information on the CBO’s location of operation, operational years, registration status, number of full-time and part-time staff and volunteers, presence of PLHIV among staff and volunteers, CBO’s social media platform usage, target client populations and routine services provided.

#### Service provision during COVID-19

questions included CBO’s operational modes during COVID-19, presence of operation interruptions during the pandemic, CBO’s target client populations during the pandemic, types of services requested and provided during the pandemic. The survey also asked about the ways clients contacted CBOs during the COVID-19 lockdown.

If CBOs reported unresolved requests or an interruption in service provision during COVID-19 lockdowns, they were asked to provide follow-up information on the types of such requests in open-ended short answers, whether service provision had resumed, and whether there was staff loss. Finally, the CBOs were asked to rate their satisfaction on their collaborations with government agencies and other CBOs during the pandemic.

#### Needs and organizational capacity building

questions started with a description of the CBO’s operational advantages and challenges during COVID-19, including short answers on CBOs’ most urgent needs, the relative advantages of their organizations compared to other CBOs, and whether their services were better recognized and expanded during COVID-19. They also estimated whether COVID-19 was helpful or not, in terms of future prospects of the organization.

### Focus group interview

While the survey focused on the challenges CBOs faced during the lockdown, a focus group interview was set up to help researchers contextualize the findings of the survey and to collect policy recommendations to support CBOs’ work in future outbreaks. All 29 survey participants were invited to the focus group interviews and eighteen CBOs (62%) participated through Tencent Meeting (an online meeting platform in China) after they completed the survey. The focus group interview was led by a Ditan Hospital HIV specialist and an HRR staff following a semi-structured question guide. The semi-structured question guide was developed to capture the following themes: (a) what services delivery challenges they encountered during COVID-19 in 2020; (b) which special groups of patients (e.g., elderly, people with disability etc.) came to their service during the COVID-19 lockdown; (c) what innovation and lessons they have learned from providing services for PLHIV during the COVID-19 pandemic; and (d) what policy recommendations do they have to enhance CBOs’ service delivery in future pandemics.The online focus group interview lasted two hours until data saturation was achieved. Detailed field notes were used for data analyses.

### Data analysis

Online survey data was collected and managed through the online service platform Wen-Juan-Xing and analyzed using STATA 17.0 [32]. We first provided descriptive statistics to summarize the characteristics of the CBOs operations, services, and clients during COVID-19 with multiple choice questions. We then used thematic analysis to analyze and explore potential themes of the open-ended questions. Two independent researchers followed the analytic process recommended for thematic analysis [33]: (a) familiarizing themselves with the data; (b) generating-e initial themes and codes; (c) coding the open-ended answers according to the themes; (d) discussing the differences, obtaining consensus, and finalizing the name of the themes; and (e) producing the report for the results section. The same approach was used to summarize additional themes from the notes of the focus group interview. Quotations were used to highlight the findings. Both survey and focus group scripts were analyzed in Chinese and translated to English for the report. Back translations were used to check for translation accuracy.

## Results

### Characteristics of CBOs

29 CBOs participated in the study and their characteristics aree summarized in Table 1. More than half (55.2%) were registered with the Chinese government as a civil organization, while 13 CBOs (44.8%) were affiliated with hospitals or the local public health agencies. More than one third of the CBOs (41.4%) in the study were located in the North or Northeast Region of China, about another third (34.5%) were from Eastern or Southern China, and seven (24.1%) CBOs were from West China. Number of staff was another measurement of the operation scale: 41.4% (n = 12) of CBOs reported more than 50 staff, while only two (6.9%) reported having less than 10 staff (Table 1). 69.0% of CBOs employed PLHIV, and all organizations reported having PLHIV among their volunteers.

**Table 1:** Select operational characteristics of CBOs serving HIV/AIDS patients in China (n = 29)

	N	%
**Years in service**		
14 yrs+	6	20.7
10–14 yrs	10	34.5
5–9 yrs	11	38.0
< 5 years	2	6.9
**Registration Status**		
Civil governance registered CBO	16	55.2
CBO affiliated with hospitals and CDC	13	44.8
**Region**		
Northeast/North	12	41.4
East/South	10	34.5
West	7	24.1
**Population of City**		
Small (0–5 million)	6	20.7
Medium (5.01-10 million)	10	34.5
Large (10.01-15 million)	7	24.1
Mega (More than 15 million)	6	20.7
**Size (#of staff and volunteers)**		
Small (0–10)	2	6.9
Medium (11–50)	15	51.7
Large (More than 50)	12	41.4

### CBOs’ new services during the COVID-19 lockdown

Even before COVID-19, Chinese CBOs often served a diverse client population. In our study, the majority (96.4%) of CBOs reported providing services to MSM, 80% catered to PLHIV, 60.7% provided services to adolescents at risk, and others facilitated services to migrants (28.6%), female commercial sex workers (25%), substance users (7.1%), children affected and orphaned by AIDS (14.3%), and the general public (25%) before the COVID-19 pandemic (Table 2). During COVID-19 lockdowns, almost all CBOs reported providing new services to non-local clients seeking HIV related services when they could not go back to their routine medical services (n = 28, 96.6%), and to their regular clients who got stranded in other cities (n = 26, 89.7%), in addition to their regular local clients. These new services were requested through various channels, including peer referrals, online platforms and group chats, hospitals and clinics, and through CBOs’ pre-pandemic services.

**Table 2:** Characteristics of client populations, services, and service platform pre- and post- COVID-19 among CBOs serving PLHIV in China (n = 29)

	N	%
**Target Client Populations- pre-COVID-19**		
MSM	28	96.6
PLHIV	23	79.3
Adolescents	18	62.1
Injection drug users (IDUs)	3	10.3
Female commercial sex workers	9	31.0
Migrants	8	27.6
General public	7	24.1
Children affected and orphaned by AIDS	4	13.8
Other	1	3.5
**New Clients During COVID-19**		
Routine local clients	27	93.1
Non-locals staying in the area due to pandemic	28	96.6
Routine clients stuck in other areas due to pandemic	26	89.7
**Routine Service Provided pre-COVID-19**		
Rapid HIV screening	27	93.1
Health education and promotion	24	82.8
Medical related care	19	65.5
Peer support	17	58.6
Community capacity building	11	37.9
Legal aid	6	20.7
Fundraising	4	13.8
Other	1	3.5
**New Services Provided During COVID-19**		
Mailing medications	26	89.7
Consultation after high-risk behaviors	25	86.2
Post-exposure prophylaxis provision	24	82.8
Online HIV counseling and testing	22	75.9
Peer support	20	69.0
In-person rapid HIV screening	19	65.5
Access to healthcare support	19	65.5
Family notification counseling	9	31.0
Other	2	6.9
**Media Platform Usage**		
WeChat	26	89.7
Website	10	34.5
TikTok	3	10.3
Other	12	41.4
**Clients’ Needs During COVID-19**		
Obtain HIV medication	28	96.6
Access timely health care	20	69.0
Counsel after high-risk behaviors	22	75.9
Peer support	21	72.4
HIV testing and counseling	19	65.5
HIV status confirmation	18	62.0
Family relationship counseling	14	48.3
Other	3	10.3
**Service Delivery Methods During COVID-19**		
Hybrid (in person and remote)	23	79.3
Remote only	6	20.7
**Clients contact methods**		
Referral from friends	29	100
Referral from WeChat groups	26	89.7
Referral from public online platforms	25	86.2
Referral from other CBOs	19	65.5
Referral from CDC or specialized hospitals	16	55.2
CBO’s routine service	16	55.2
Brochures	6	20.7
Other	3	10.3

The scope of CBO services also changed during the COVID-19 lockdown period. While most CBOs reported continued provision of regular services, they had to add services unique to the lock-down period, including mailing ART medicines and post-exposure prophylaxis (PEP), supporting family notification of PLHIV’s HIV status, and peer support (Table 2).

Not only did the service requests intensify during the COVID-19 lockdown, but many CBOs also had to switch their on-site services to phone- and online-based when the travel restrictions hit. All participating CBOs reported using one or more new platforms such as WeChat, TikTok, etc. for services during this period (Table 2). Meanwhile, most CBOs still maintained on-site services except for five who stopped providing in-person services.

To cope with the challenges brought by the pandemic, many CBOs turned to innovative communication strategies and digital technology to secure medicine supplies. One CBO said: *“There are 10 internet volunteers to promote online, and 5 volunteers to make appointments for testing, so that you can ensure access to various HIV supplies via the internet without having to come to the office.”* (Medium CBO, 5–9 yrs of operation, independent, large city in the West). Six CBOs also attributed human-centered care and respect for PLHIV, their rapport with marginalized clients, and their ability to operate at the grass-roots level as key factors in their success. As one CBO put it: *“Government agencies work according to rules and regulations, while friends of the community work more on the basis of their enthusiasm and human feelings for the community.”* (Large CBO,10–14 yrs of operation, independent, small city in the Northeast/North).

### Challenges and coping strategies during COVID-19

While many CBOs successfully carried out services for PLHIV, challenges still mounted during the unexpected lockdowns. In the survey, most CBOs (90%) reported unmet needs from their clients during the pandemic, especially on access to ART (41%), referral to health care (24%), and HIV testing and confirmation (21%). About a third of CBOs reported that their services were disrupted by the pandemic, particularly their in-person services (31.0%). Among the services most impacted were in-person HIV testing (27.6%), in-person counseling (10.3%), in-person volunteering (3.4%) and outreach (3.4%). After the lockdown, all CBOs resumed their services. However, 33.3% of organizations reported losing staff.

When asked about the top three challenges during the COVID-19 lockdown, 18 CBOs identified limited service provision modes as their main challenge, followed by shortage of personal protective equipment (PPE) for staff (51.7%), lack of funding (37.9%), staff shortages and loss of staff and volunteers (17.2%), limited or slow HIV testing services (10.3%), lack of support from other sectors and society at large (3.4%), limited medical resources (3.4%), and hard to obtain and deliver medication (13.8%). CBOs also mentioned short-term funding shortfalls, weak Wi-Fi, and inaccessibility of office space as challenges.

One additional challenge CBOs reported was the lack of mail courier services, especially in remote areas and to college students who live on locked-down campuses. One CBO observed: *“Some patients stop taking their medication when they can’t even get to the courier company in places where transportation is inconvenient.”* (Medium CBO, 5–9 yrs of operation, civil governance registered, medium city in the West). Another said: *“It is difficult to give medication to students because the school is very strict, no deliveries to campus for 8 months, and security checks to enter school, risking exposure of privacy.”* (Medium CBO, 5–9 yrs of operation, independent, small city in the West).

Another common challenge was associated with HIV testing and confirmation tests: *“There has been no opportunity to confirm and then start treatment for infected persons with positive initial screening due to the lockdown and home isolation…. Someone tested positive in July and it took three months to confirm.”* (Medium CBO, 5–9 yrs of operation, independent, small city in the West).

Finally, several CBOs mentioned that they could not meet clients’ needs in gaining peer support, resolving financial issues and how to navigate other barriers.

An important coping strategy among the CBOs was building collaboration with various government agencies, including local CDC (86.2%), hospitals (86.2%), the Ministry of Public Security (10.3%), and local government organizations or other agencies (10.3%). On a scale from 0 to 10, most organizations were very satisfied with their collaborations during the pandemic (24 CBOs > 8), four CBOs were somewhat satisfied (ratings of 5–7), and only one CBO was completely unsatisfied (rating of 0). Almost 80% of CBOs reported collaborations with other non-governmental organizations, mostly with similar organizations in the same region (e.g., provincial and municipal sister agencies). Such collaboration helped relieve shortages of service, medicine, and PPEs.

### CBO’s sustainability and capacity building needs

When asked about the top three areas of needs, 15 CBOs named funding and personnel as their top need (51.72%). Other top needs included technical assistance, supplies, organizational capacity building, and collaboration among CBOs and with other sectors. While the second and third needs varied, organizational capacity building was mentioned seven times (24.14%), demonstrating its significance for the CBOs. Only two CBOs did not report any external needs.

Many organizations felt that the capacity needed during the pandemic were better coordination and communication skills (27.6%), flexibility (20.7%), and good service provision (13.8%). Six CBOs also emphasized that the ability to provide online consultation and telemedicine services were useful skills during a pandemic. A large network and emergency response training were also desirable capacities reported by the CBOs.

Despite the many challenges faced by the participating CBOs and their clients during the COVID-19 pandemic, most of the CBOs felt that they had gained popularity during the pandemic, and many mentioned thank-you-notes from their clients. They were also able to build a larger network with requests from other organizations (34.5%), received media coverage (20.7%), and additional project funding (31.0%) during the pandemic. About half of the CBOs considered the COVID-19 pandemic beneficial to their organization’s development.

### Policy recommendations from the CBOs

The focus group interview helped confirm capacity-building needs. More importantly participating CBOs also offered the following recommendations during the focus group discussion, which expand on the needs expressed in the survey. We summarized the policy recommendations in the following:

- **Create regional CBO service alliance networks**: *“Alliances can be formed between community organizations, and a directory of information on local drug assistance, etc., can be produced and sent to patients so that they can refer to the directory for targeted help.”* (Large CBO, 5–9 yrs of operation, independent, mega city in the Northeast/North).
- **Strengthen multisectoral cooperation** between CBOs, hospitals, the CDC, community level government and community health centers from different regions: *“… the local CDC alone may not be able to get the job done. There is a need for the creation of a network of emergency support services in the event of an emergency.”* (Large CBO, 14 yrs + of operation, civil governance registered, large city in the Northeast/North).
- **Develop standardized emergency response operational manuals/guidelines** (including recruitment). One CBO talked about preparing both CBOs and their clients by establishing guidelines: *“There should be guidelines for patients, but also for community organizations, including what community organizations can do, how to do it, how to do risk assessment (e.g. group lending, mutual aid lending), and what are the channels for obtaining supplies; for patients, it is a manual for self-management of infected persons in case of emergency.”* (Large CBO, 14 yrs + of operation, civil governance registered, large city in the Northeast/North).
- **Support PLHIV community resilience building**. Many CBOs discussed ways to build resilience among PLHIV during emergencies, including on ART: *“Infected people need to be better guided and educated about treatment adherence.”* (Small CBO, 5–9 yrs of operation, independent, medium city in the East/South).
- **Optimize medicine access during a crisis**. CBOs identified access to medicine as the top challenge during COVID-19 and the necessity of flexible policy on longer-term prescriptions: *“It would be better for the infected to be prepared when something like this happens again, and to have peace of mind, if they are advised by the agency that dispenses the drugs.”* (Large CBO,5–9 yrs of operation, independent, medium city in the East/South).
- **Build a larger volunteer pool to offset staff shortage during a crisis**: *“Policies are needed to involve volunteers in the fight against the pandemic and AIDS.”* (Medium CBO, 5–9 yrs of operation, independent, small city in the West).

## Discussion

COVID-19 posed unprecedented challenges to global health and reversed decades of hard-earned progress on health [34]. Our study was one of the first to survey and interview frontline CBO staff that serve PLHIV around China in the first year of the pandemic. The CBOs in our study varied in their operational size and their affiliations status with the CDC and clinics. While some work in urban centers with advanced economic development, others are located in rural districts. In both the survey and the focus group interview, the common themes were the challenges they faced unexpectedly when COVID-19 hit and how the strict lockdown added to their service scope and forced them to change service methodology. Our survey findings add to the growing literature on resilience of communities during a natural disaster and highlight the importance of networking, digital platforms, and operational flexibility among grass-root community organizations. The focus group interview with the CBOs further explained the mechanisms of coping and provided a much-needed reflection on the need for capability building for future pandemics or disasters.

Our study found that CBOs serving people living with HIV in China often had at least 5–10 years of experience working with local PLHIV and generally have good working knowledge and close collaborations with local public health agencies and medical institutions. This type of three-in-one network has proven to improve performance metrics on disease testing and detections among high-risk populations [35]. The community organizations also serve a diverse population, including PLHIV as well as people at higher risk for HIV, such as MSM and female sex workers, and routinely carry out services including health promotion, peer support, and treatment coordination. Many CBOs have volunteers from the local PLHIV or high-risk population, which enables them to conduct targeted outreach and build trust with their clients. This finding validates a community resilience theory [36] that named four primary sets of adaptive capacities as critical to community resilience: social capital, economic development, information and communication, and community competence. The social capital, community competency, and communication skills of the CBOs have helped the organizations to achieve high efficiency in HIV control with flexible working venues and low operating costs.

During emergencies, to build collective resilience, communities must create organizational linkages, boost and protect social supports, and plan for not having a plan – which requires flexibility, decision-making skills, and trusted sources of information that function in the face of unknowns [36]. Our study found that CBOs that can provide PLHIV with wider organizational connections and mobilize social support through flexible operation plans, had a pivotal role in building community resilience during COVID-19. For example, despite the fact that the National Center for AIDS/STD Control and Prevention of China’s CDC issued a special policy to facilitate ART treatment continuity among PLHIV at the early stage of the COVID-19 pandemic [37], travel restrictions under the COVID-19 lockdown still had a significant impact on PLHIV’s access to and the CBO’s ability to provide services, such as ART, testing, and other medical care [13]. Since the lockdown happened at Chinese New Year, a time when internal migration is at its peak and a large population visits their family away from where they work, many PLHIV found themselves stuck in places away from their routine care. Many who had to quarantine with their parents found themselves having to forgo privacy and to disclose to their parents and families for the first time that they needed HIV care [27, 38]. Meanwhile, most of the local staff at public health stations were redeployed to respond to COVID-19. CBOs found significant workload increase with new clients stranded in their location in need of HIV care, new demands to coordinate HIV treatment continuity and testing, and additional requests on peer support in this time of crisis. In response, many CBOs had to make quick decisions to switch their service online and to seek new connections with fellow organizations and new government agencies– with varying success as our survey showed. Similar to our study’s findings, a 2020 China AIDS Fund for NGOs and UNAIDS’s CBO survey found that 87% of community organizations set up their own hotlines and implemented flexible working hours for volunteers during the COVID-19 epidemic from February to April 2020, to provide AIDS-related services. Nearly half added express mailing services for delivery of HIV self-testing kits and medicine to their services [39].

The same UNAIDS survey revealed that some CBOs encountered difficulties in HIV/AIDS services during the peak of the pandemic during February and March of 2020. The main reasons for the service interruption were urban traffic control (86%) and CDC staff who were unable to support AIDS prevention work (51%) because of their participation in the prevention and control of the COVID-19 epidemic. By the end of April 2020, about 53% of community organizations had fully restored their services, and 42% had restored some services [39]. Our findings are in line with the scenario described in the UNAIDS report and found the most critical challenges to the CBOs to be a lack of funding, limited service provision methods, shortage of staff and PPE, limited medical resources, testing and medication delivery capacities, and lack of support and understanding from society at large. All these factors contributed to CBOs’ service disruption, in addition to the reasons identified by the UNAIDS survey.

Paton (2000) defined community resilience as the capacity to bounce back and use physical and economic resources effectively to aid recovery following exposure to hazards [40]. In an earlier report for UNAIDS, we found that as a vulnerable population, PLHIV faced unique challenges during the unexpected COVID-19 lockdowns [41]. However, they had better resiliency resources – the CBOs that had already served their community before the pandemic – compared to other vulnerable populations, such as migrant workers and people with disabilities in China [41]. To build stronger resilience in the face of future disaster situations, the CBOs in our study offered multiple suggestions that we can summarize into four recommendations: First, enhance CBOs network building both horizontally and vertically: horizontally among CBOs with similar missions across different geographic areas so that when PLHIV travel, they can rely on the network for the continuation of support; vertically between CBOs and multiple hierarchies of government and healthcare facilities for resource coordination. Such collaborations should be included in government-level emergency response plans and policies to ensure the continuation of support for PLHIVs.

Secondly, funding agencies should consider supporting CBOs’ capacity building in communication and technology upgrades so that CBOs can expand their digital direct service platforms and mobilize resources during a crisis.

Thirdly, CBOs should consider strengthening their volunteer base and building a workforce reserve for their community-based services to prepare for staff shortages during emergencies. CBOs can mobilize these local talents from various groups. One way is to empower PLHIV to become peer supporters/volunteers during emergencies. Their presence in an emergency response would benefit the utilization of community-based services, and reduce PLHIV’s unease to disclose their status and seek help. Another way to engage more talents to grassroot community governance is to provide CBO-based internships for college students and professional training institutes (e.g., to students of public health, medicine, sociology, or other related fields). Efforts should also be made to cultivate professional talent serving grassroot communities, adjust policies and incentive mechanisms, and encourage more experts to provide intellectual support for community governance. Encouraging the public to participate in grassroots social governance can also help empower community members and generate increased cohesion and community resilience.

Finally, the CBOs’ capacity building should include a community-level emergency response plan. Education on disaster prevention and mitigation at the community level should be strengthened, and communities with excellent emergency response and disaster relief operations should be promoted as models, so their experiences can be shared. Bureaucratically and institutionally, the division of labor in emergency response and community governance should be further clarified, and community staff should receive training in emergency planning and response, to provide speedy and efficient public service when faced with future uncertainties and emergencies.

The findings of this study should be viewed in the context of several limitations. First, the study survey was cross-sectional. Therefore, causal inference could not be established, and the results can only reflect the situation of CBOs during a certain period during the pandemic. Second, the study only analyzed 29 responses from different CBOs and their operational branches, utilizing the connections of HRR. The limited number of participants and the recruitment method may have led to biased results, as the selected CBOs might not be representative of the population. Third, all information in the study was self-reported by one manager of each CBO, thus is subject to risk of bias.

## Conclusion

Risks and vulnerabilities induced by pandemics and other natural hazards and disasters are on the rise globally. Some emergencies, such as COVID-19, have severe and widespread destructive impacts on health, the economy, social development, and global supply chains. In this context, community resilience to disasters is critical for government hazard mitigation and recovery planning [36, 42]. Our case study showed that CBOs serving a highly stigmatized and vulnerable population before the crisis were instrumental in building resilience in the community. They were able to quickly mobilize resources, set up new business platforms/models, and expand their network to meet unprecedented challenges. They also identified key areas for capacity building for future crisis preparedness. Their experience and reflections may help governments, communities, and international organizations when considering how to reduce health inequity and how to serve those who need long-term healthcare during an unexpected natural or manmade crisis.

## Data Availability

The datasets used and/or analyzed during the current study are available from the corresponding author upon reasonable request.
